# Impact of Blood Pressure Levels During Pregnancy on Postpartum Hypertensive Outcomes: Insights from a Cohort Study

**DOI:** 10.3390/jcm15124646

**Published:** 2026-06-15

**Authors:** Anne-Christin Loheit, Charlotte Lößner, Yvonne Lindemann, Ekkehard Schleussner, Tanja Groten

**Affiliations:** 1 Department of Obstetrics, University Hospital Jena, Friedrich-Schiller-University Jena, Am Klinikum 1, 07747 Jena, Germany; 2 Department of Gynecology, University Hospital Jena, Friedrich-Schiller-University Jena, Am Klinikum 1, 07747 Jena, Germany; 3 Department of Obstetrics, University of Cologne, Faculty of Medicine and University Hospital Cologne, 50931 Cologne, Germany

**Keywords:** blood pressure management, pregnancy, preeclampsia, fetal growth restriction, postpartum hypertension, cardiovascular risk

## Abstract

**Background/Objectives:** This cohort study aims to evaluate the association between antenatal blood pressure levels and postpartum cardiovascular outcomes in women with preeclampsia and/or fetal growth restriction (FGR). The objective of the study was to test the hypothesis that blood pressure levels during pregnancy are associated with cardiovascular health after delivery. **Methods:** The study was conducted at a tertiary hospital in Germany and involved women who developed preeclampsia and/or FGR during their pregnancies between 2021 and 2024. Participants were invited for cardiovascular follow-up consultations at 6 weeks and 6 months postpartum. VICORDER measurements were used for cardiovascular function assessment. Results were compared between the group with persisting hypertension and normotensive women at each study visit to assess the long-term progression of hypertensive disorders in relation to antenatal blood pressure levels. Statistical analysis employed Mann–Whitney U tests and adjusted odds ratios (aORs). **Results:** Of the 103 women who attended postpartum cardiovascular consultations during the study period, a substantial proportion still had elevated blood pressure at 6 weeks (51.49%) and 6 months (42.86%) after delivery. Women with persistent hypertension had higher systolic and diastolic blood pressure throughout pregnancy (*p* < 0.001), used antihypertensive medication more frequently (*p* < 0.001) and showed significantly increased arterial stiffness up to 6 weeks postpartum compared with normotensive women (7.4 m/s vs. 6.7 m/s, *p* < 0.001). In adjusted analyses, higher blood pressure levels during pregnancy were significant predictors of persistent postpartum hypertension (*p* < 0.05). Except for systolic blood pressure in the 3rd trimester at the 6-week follow-up, every 10 mmHg increase in blood pressure during pregnancy was associated with higher odds of postpartum hypertension, with aORs ranging from 1.82 to 3.42 at 6 weeks and from 2.18 to 5.51 at 6 months postpartum (depending on the trimester). **Conclusions:** Maintaining healthy blood pressure levels during pregnancy may reduce the long-term cardiovascular risk for mothers. These findings underscore the importance of early detection and consistent management of hypertension in expectant mothers with hypertensive pregnancy disorders. Further research is required to identify optimal blood pressure management strategies during pregnancy that could improve immediate and long-term maternal health outcomes.

## 1. Introduction

Hypertensive disorders represent some of the most frequent complications of pregnancy, with a global prevalence of 5.2–8.2% [1], and have considerable implications for both maternal and neonatal health. Effective blood pressure management during pregnancy is essential to reduce the risks associated with hypertensive disorders [2,3]. A study by Tita et al. demonstrated that pregnant women with mild chronic hypertension who received prompt treatment to maintain blood pressure below 140/90 mmHg had improved pregnancy outcomes compared to those who delayed treatment until severe hypertension developed (systolic blood pressure above 160 mmHg and/or diastolic blood pressure above 105 mmHg) [3]. Importantly, this approach did not result in a higher rate of small-for-gestational-age births. Hypertensive disorders during pregnancy are linked to accelerated cardiovascular aging in the affected women, who are at increased risk of developing coronary artery disease, heart failure, aortic stenosis, and mitral valve regurgitation later in life compared to those who remained normotensive during pregnancy [3]. Notably, one year postpartum, patients with preeclampsia have a higher risk for heart failure [4], and 41.5% still have high blood pressure [5]. Despite these associations, it remains unclear how blood pressure levels during pregnancy influence the long-term cardiovascular outcomes in women with hypertensive disorders. Therefore, the present study investigated the impact of the obtained blood pressure values during pregnancies complicated by preeclampsia and/or fetal growth restriction (FGR) on the cardiovascular risk profile 6 weeks and 6 months postpartum.

## 2. Materials and Methods

### 2.1. Design and Study Population

This single-center cohort study was conducted at a tertiary care center in Germany. Women who developed preeclampsia and/or FGR during their pregnancies and delivered at our institution during 2021–2024 at a minimum of 24 completed weeks of gestation were routinely invited to a cardiovascular follow-up consultation at 6 weeks and 6 months postpartum.

According to the German national guidelines (Update 2024), preeclampsia is defined as chronic or gestational hypertension with at least one organ manifestation during pregnancy that cannot be attributed to any other cause [6]. Both singleton pregnancies and twin pregnancies are included.

To assess the long-term progression of hypertensive disorders in relation to blood pressure levels during pregnancy, we compared clinical characteristics and measures of cardiovascular function between the group with hypertension and normotensive women at the time of follow-up examination.

Hypertension was defined as a systolic blood pressure ≥ 140 mmHg and/or a diastolic blood pressure ≥ 90 mmHg, or the current use of antihypertensive medication. Normotension was defined as a systolic blood pressure < 140 mmHg and a diastolic blood pressure < 90 mmHg, without the use of antihypertensive medication. Ethical approval was obtained from the Ethics Committee of our institution (no. 2025-3972-BO_D).

### 2.2. Data Collection and Assessment of Clinical Characteristics

Women underwent a comprehensive medical history and cardiovascular status evaluation, including VICORDER measurements (80 Beats Medical, Berlin, Germany) at each postpartum visit. In addition to blood pressure readings taken during the follow-up examination, values from each trimester of pregnancy were retrieved from maternity and medical records retrospectively. For standardization, where available, the minimum and maximum blood pressure values per trimester were documented, and the mean was calculated and reported. Owing to existing pregnancy complications, many measurements were taken during ongoing antihypertensive therapy. In addition to the cardiovascular examination, information about the patients’ physical activity in the 7 days prior to the study visit was collected using the short version of the International Physical Activity Questionnaire (IPAQ-Short version) [7]. Data collection also covered pre-pregnancy chronic conditions, maternal body mass index (BMI) before conception, gestational age at delivery, child birth weight, occurrence of pregnancy complications, and medication use during pregnancy. In addition, data on diet, nicotine and alcohol consumption, and socio-economic status were collected at the study visit (see Tables 1 to 3 for the complete list of data collected). All data relating to pregnancy history and cardiovascular status were analyzed and incorporated into personalized recommendations for the patient. The data analysis for this study was conducted retrospectively. Since the data were collected as part of routine clinical care and were analyzed retrospectively, informed consent from the patient was not required for this study.

### 2.3. Hemodynamics

VICORDER measurements were conducted by a trained study nurse during the study visit, in accordance with the manufacturer’s guidelines. Participants were positioned supine with their upper body elevated at a 30° angle for these assessments. They were instructed to remain still and not to speak during the procedure. Brachial blood pressure readings were manually recorded beforehand. VICORDER analysis included parameters such as pulse wave velocity (PWV), pulse wave analysis (PWA) and flow-mediated slowing (FMS), as specified by the device. FMS measurements were carried out using a method involving upper-arm and wrist occlusion; a vacuum cushion was used to ensure stable arm positioning. Blood pressure readings were taken using non-invasive, automatic, oscillometric devices, in accordance with standard medical protocols.

### 2.4. Statistical Analysis

Mann–Whitney U tests were used to compare group characteristics. Adjusted odds ratios (aORs) were calculated to assess the risk of postpartum hypertension based on blood pressure measurements taken during pregnancy. A regression analysis was performed for both systolic and diastolic blood pressure values for each trimester. This adjustment was made for potential confounders such as maternal BMI, use of antihypertensive medication, chronic hypertension prior to pregnancy and the occurrence of pregnancy complications. The Wald backward method was used for binary logistic regression analysis. Variables with a *p*-value > 0.10 were removed from the model. To improve clinical interpretability, aORs and corresponding confidence intervals (CIs) are presented per 10 mmHg increase in blood pressure in the Section 3. Results were considered statistically significant at *p* < 0.05. Statistical analyses were performed using IBM SPSS Statistics version 29.0 (IBM Corp., Armonk, NY, USA).

## 3. Results

### 3.1. Cohort

**Figure 1:**
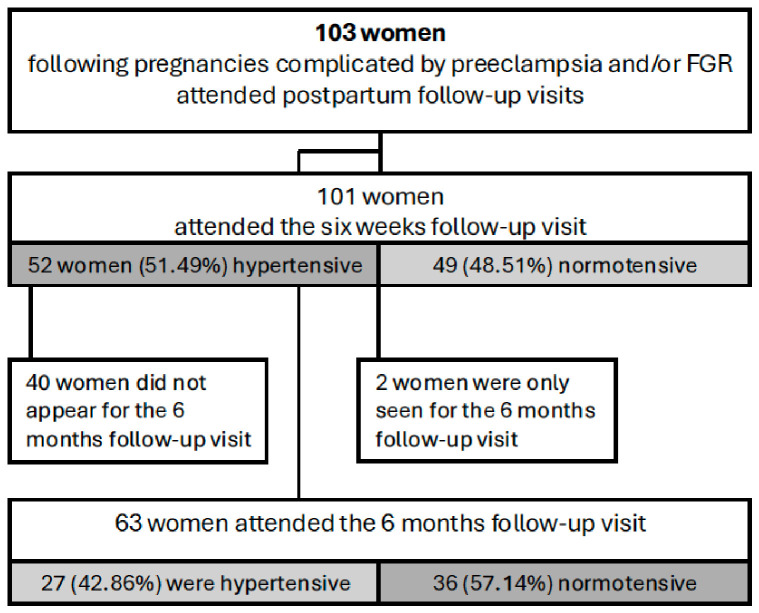
Cohort Composition.

A total of 103 patients attended a cardiovascular follow-up consultation. Of the 101 patients attending the 6-week follow-up visit, 63 were seen at 6 months. Two patients were only seen at 6 months. Of the 101 patients attending the cardiovascular assessment 6 weeks postpartum, 52 women (51.49%) were classified as hypertensive and 49 (48.51%) as normotensive. At 6 months, of the 63 women seen, 27 (42.86%) were hypertensive and 36 (57.14%) were normotensive. The subsequent analyses compared these groups (Figure 1). Of the 101 patients, 61 returned after 6 months; 39.6% of these patients attended only the first appointment, 6 weeks after giving birth. When comparing women who attended their appointment at 6 months with those who did not return 6 months postpartum, there were no differences in baseline characteristics such as BMI (*p* = 0.461), systolic blood pressure at 6 weeks (*p* = 0.160), diastolic blood pressure at 6 weeks (*p* = 0.319), parity (*p* = 0.458), smoking (*p* = 0.150), socio-economic status (highest level of education, *p* = 0.458) or dietary habits (*p* > 0.05).

### 3.2. Clinical Characteristics According to Blood Pressure Trends During Pregnancy

Clinical characteristics of women attending the follow-up visit at 6 weeks are depicted in Table 1. At 6 weeks postpartum, women with hypertension had a higher prevalence of pre-existing chronic hypertension (40.4% vs. 10.5%, *p* < 0.001) and showed higher pre-pregnancy BMI measures (26.57 kg/m^2^ vs. 23.42 kg/m^2^, *p* < 0.001). During pregnancy, women with persistent hypertension experienced a shorter duration of pregnancy (35 weeks vs. 37 weeks, *p* < 0.001), and their childs’ birth weight was significantly lower (2075 g vs. 2520 g, *p* = 0.042) (Table 1).

Throughout pregnancy, women who remained hypertensive postpartum consistently exhibited higher systolic and diastolic blood pressure values across all trimesters (*p* < 0.001). Consequently, this group required antihypertensive medication during pregnancy more frequently than those who were normotensive at follow-up (86.5% vs. 42.9%, *p* < 0.001) (Table 1).

Clinical characteristics and group comparison results of women attending the 6-month follow-up visit are depicted in Table 2. At 6 months postpartum, comparison between normotensive and hypertensive women revealed that, in addition to the previously described differences, those in the hypertensive group had significantly higher systolic and diastolic blood pressure values across all trimesters (both *p* < 0.001). Furthermore, antihypertensive medication was also significantly more commonly used by this group during pregnancy (92.6% vs. 47.2%, *p* < 0.001) (Table 2).

### 3.3. Assessment of Cardiovascular Function Using VICORDER

Cardiovascular analyses 6 weeks postpartum revealed significant differences between hypertensive and normotensive women. Those with persistent hypertension exhibited higher peripheral pulse pressure (PP) (42.5 mmHg vs. 38 mmHg, *p* = 0.010), aortic pulse pressure (AoPP) (40.5 mmHg vs. 36.25 mmHg, *p* = 0.007), and aortic systolic blood pressure (AoBP sys) (129.75 mmHg vs. 111.75 mmHg, *p* < 0.001) (Table 3). Pulse wave velocity (PWV) was also significantly greater in the hypertensive group (7.4 m/s vs. 6.7 m/s, *p* < 0.001), as was cardiac output (CO) (4.89 L/min vs. 4.28 L/min, *p* = 0.010) (Table 3).

At 6 months postpartum, hypertensive women continued to show higher values for AoBP sys (128 mmHg vs. 116.25 mmHg, *p* < 0.001) compared to normotensive women (Table 3). Mean arterial pressure (MAP) was also significantly elevated (110 mmHg versus 98.5 mmHg, *p* < 0.001), as was total peripheral resistance (TPR) (1.39 PRU versus 1.14 PRU, *p* < 0.001). Cardiac index (CI) values were higher in normotensive women than in hypertensive women (2.9 L/min/m^2^ versus 2.48 L/min/m^2^, *p* = 0.013) (Table 3).

In the multivariable analysis, models were adjusted for pre-pregnancy BMI, use of antihypertensive medication during pregnancy, pre-existing chronic hypertension, and pregnancy complications (preeclampsia and/or fetal growth restriction). Higher blood pressure levels during pregnancy, except the 3rd trimester systolic blood pressure at 6 weeks follow-up, were independently associated with an increased likelihood of persistent postpartum hypertension at both time points. Except for systolic blood pressure in the 3rd trimester at the 6-week follow-up, every 10 mmHg increase in blood pressure during pregnancy was associated with higher odds of postpartum hypertension, with aORs ranging from 1.82 to 3.42 at 6 weeks and from 2.18 to 5.51 at 6 months postpartum (depending on the trimester). All models had a good to very good explanatory power (Nagelkerke’s R-squared) (Table 4). The final step for each regression analysis, including all additional factors, can be found in the Supplementary Materials.

## 4. Discussion

### 4.1. Principal Findings

The study shows that blood pressure levels during pregnancy may affect maternal blood pressure up to at least 6 months postpartum. Using the recorded blood pressure values for each trimester, our multivariable analysis demonstrated that higher values across pregnancy (with the exception of the systolic values in the 3rd trimester at 6 weeks follow-up) were consistently associated with an increased likelihood of hypertension at both 6 weeks and 6 months after delivery.

### 4.2. Results in the Context of What Is Known

Accompanying analyses of VICORDER measurements showed that women with persistent hypertension had significantly higher vascular stiffness up to 6 weeks postpartum, and a milder, only trend-level increase at 6 months. Based on the 2010 reference values for pulse wave velocity (PWV) from “The Reference Values for Arterial Stiffness’ Collaboration” in 2010 [8] (median PWV 6.5 m/s for women in their thirties), these findings suggest that women without persistent hypertension return more quickly to age-appropriate PWV levels. However, this analysis did not examine PWV values during or prior to pregnancy, so it cannot be ruled out that the differences were already present beforehand.

The present results show a significantly lower CI in hypertensive women compared with normotensive women at the 6-month follow-up (2.48 vs. 2.9 L/min/m^2^, *p* = 0.013). This finding appears counterintuitive at first glance, as the early phase of hypertension is typically characterized by a hyperdynamic state with an elevated or normal CI [9]. However, there are two main pathophysiological and methodological explanations for this discrepancy: Firstly, this could indicate persistent vascular dysfunction and cardiac remodeling. While acute hypertension is often volume-driven, chronic pressure overload may lead to concentric myocardial hypertrophy and increased left ventricular stiffness (diastolic dysfunction). This may restrict ventricular filling and reduce stroke volume, which could contribute to a reduced CI despite the increased afterload resistance [10]. Secondly, the influence of antihypertensive medication must be taken into account. A blockade of beta-adrenoceptors (beta-blocker therapy) directly reduces the heart rate and the contractile force of the myocardium [11]. This study did not record the patients’ specific antihypertensive treatment regimens. However, beta-blockers are part of the standard treatment for hypertension in Germany. If a significant proportion of the hypertensive cohort had been treated with beta-blockers, this iatrogenic reduction in cardiac output may have contributed to the lower CI.

Hypertensive women also showed signs of sustained blood pressure load, with higher mean arterial pressure at both follow-up visits and increased pulse pressure at the 6-week postpartum visit, which was no longer apparent at 6 months. Elevated pulse pressure is not only a marker of cardiovascular risk but also actively contributes to adverse outcomes such as myocardial infarction, heart failure, and cardiovascular death, even in individuals with otherwise normal blood pressure [12]. Total peripheral resistance was elevated in both groups but was substantially higher in hypertensive than in normotensive women, consistent with impaired endothelial function and increased arterial stiffness in the context of persistent hypertension. Our findings contribute to the existing body of research on blood pressure levels during pregnancy and highlight the potential importance of consistent blood pressure monitoring and structured postnatal care. In particular, high-risk groups benefit from close monitoring and follow-up in the long term [2,3,4,13]. Effective postpartum monitoring and management are essential to mitigate cardiovascular risk and reduce the incidence of secondary complications such as coronary heart disease and heart failure, which often manifest clinically within the first year after delivery [4]. In a study by Riemer, it has been shown that PWV can be significantly reduced through dietary intervention in combination with a 6-month intensive cardiovascular training program [14]. However, we collected data on physical activity using the International Physical Activity Questionnaire (IPAQ). Results revealed that there was no difference in physical activity reported between the groups (Table 1 and Table 2).

### 4.3. Clinical Implications

In 2022, guideline recommendations for blood pressure control during pregnancy were revised to lower targets, with a diastolic goal of 85 mmHg and a systolic goal of less than 130 mmHg [15]. However, the implementation of new guidelines into clinical practice often takes time. As a result, insufficient management of hypertensive disorders during pregnancy may contribute to long-term hypertension and cardiovascular disease in affected women. Our study provided insights into such associations. Our data may suggest that the new guidelines, which call for stricter control of blood pressure levels during pregnancy, could be beneficial. Although it remains unclear whether the cardiovascular markers identified in our postpartum assessment represent complications arising from pregnancy itself or reflect pre-existing, previously unrecognized cardiovascular disease in women who developed preeclampsia, our findings underscore the critical importance of comprehensive cardiovascular risk assessments alongside consistent blood pressure control within the targets. In addition, our study demonstrates the importance of structured after-care programs providing follow-up for this high-risk group.

Furthermore, our data demonstrate that, even in a setting providing a close personal relationship with the patients cared for, we lost about 40% of the women between the 6-week and the 6-month follow-up. Strategies to improve follow-up care adherence are needed. Comprehensive postpartum care following hypertensive pregnancies is essential not only for detecting elevated cardiovascular risk but also for guiding timely lifestyle interventions, early pharmacological treatment, and ongoing follow-up by multidisciplinary teams when indicated.

### 4.4. Research Implications—Unanswered Questions; Proposals for Future Research

Future research should aim to refine strategies for blood pressure control during pregnancy. In particular, individualized antihypertensive approaches tailored to the underlying hemodynamic profile may offer an opportunity to optimize maternal blood pressure regulation and potentially improve long-term cardiovascular outcomes [16]. At the same time, greater attention should be directed toward identifying women with hypertensive disorders of pregnancy who carry an elevated cardiovascular risk and would benefit from structured and intensified postpartum surveillance. Future studies should investigate whether vascular stiffness assessment in women with hypertensive pregnancy disorders—especially those with persistent postpartum hypertension—provides incremental value for individualized cardiovascular risk stratification beyond conventional clinical risk markers.

### 4.5. Strengths and Limitations

This study collected real-world data from women who were invited to postnatal follow-up appointments at the university hospital following preeclampsia and/or FGR. This naturally means that only women who attended these appointments were included. This could be a limitation, as it suggests that not all women with a history of preeclampsia were included, but rather only those who are likely to be more attentive to their health. In addition, we had varying numbers of blood pressure readings for the women during pregnancy, as the data were collected retrospectively. It was therefore decided to take the average of the minimum and maximum blood pressure readings recorded for each patient in each trimester. This approach may introduce measurement bias.

Because detailed information on antihypertensive treatment regimens was not systematically collected, we were unable to clearly differentiate the impact of disease severity from the effects of antihypertensive therapy.

The non-attendance rate at the 6-month follow-up appointment is high. However, no significant differences in baseline characteristics were found between the women who attended the 6-month follow-up appointment and those who did not. A bias resulting from study withdrawal cannot be ruled out.

Despite the limited sample size and the retrospective nature of our study, our real-world data provide valuable clinical insights and add to the growing body of evidence supporting careful blood pressure control and cardiovascular monitoring during and after pregnancy.

## 5. Conclusions

Our findings suggest that lower blood pressure levels during pregnancy may lower the mother’s long-term risk of developing cardiovascular disease. This highlights the critical importance of preventive measures and the early detection of hypertension in expectant mothers as a prerequisite for timely intervention and consistent blood pressure control within the targets. Future research should focus on prospective studies investigating the most effective timing and approaches for blood pressure management during pregnancy, with the aim of improving immediate and long-term maternal health outcomes. Additionally, investigating tailored treatment options for pregnant women at higher risk of hypertension is an important area for future studies.

## Figures and Tables

**Table 1:** **Group characteristics 6 weeks postpartum**.

	Women with Hypertension ^1^ (*N* = 52)	Women Without Hypertension ^1^ (*N* = 49)	*p*
**Group Characteristics Prior to Pregnancy**			
Chronic diseases prior to pregnancy			
Arterial hypertension ^2^	21 (40.4), *N* = 52	5 (10.2), *N* = 49	**<0.001**
Other Diseases ^3^	24 (46.2), *N* = 52	17 (34.7), *N* = 49	0.205
**Group characteristics during pregnancy**			
Maternal Age at delivery ^(years)^	33 (29–36), *N* = 52	32 (29–35), *N* = 47	0.507
Maternal BMI prior to pregnancy ^(kg/m^2^)^	26.57 (23.39–35.36), *N* = 49	23.42 (20.63–25.61), *N* = 49	**<0.001**
Weeks of gestation ^(weeks)^	35 (32–37), *N* = 51	37 (35–38), *N* = 47	**<0.001**
Child birth weight ^(g)^	2075 (1300–2685), *N* = 47	2520 (1875–2921.25), *N* = 42	**0.042**
Twin Pregnancies	4 (7.7), *N* = 52	0 (0), *N* = 49	
Presentation of pregnancy complication PE with FGR	23 (44.2), *N* = 52	14 (28.6), *N* = 49	0.104
Presentation of pregnancy complication PE without FGR	24 (46.2), *N* = 52	16 (32.7), *N* = 49	0.168
Presentation of pregnancy complication FGR without PE	5 (9.6), *N* = 52	19 (38.8), *N* = 49	**<0.001**
Medication intake during pregnancy			
Antihypertensive medication	45 (86.5), *N* = 52	21 (42.9), *N* = 49	**<0.001**
BP during pregnancy			
BP systolic 1st trimester ^(mmHg)^	130 (122.25–140), *N* = 38	115.5 (105.5–127) *N* = 35	**<0.001**
BP diastolic 1st trimester ^(mmHg)^	85 (80–95), *N* = 38	71.5 (67.5–80), *N* = 35	**<0.001**
BP systolic 2nd trimester ^(mmHg)^	135 (126.5–144.75), *N* = 49	111.25 (106.5–130), *N* = 42	**<0.001**
BP diastolic 2nd trimester ^(mmHg)^	87.5 (80–92.75), *N* = 49	73.25 (65–81.5), *N* = 42	**<0.001**
BP systolic 3rd trimester ^(mmHg)^	139.75 (130.5–147.5), *N* = 40	127 (113.25–139.63) *N* = 46	**<0.001**
BP diastolic 3rd trimester ^(mmHg)^	87.75 (82.75–93), *N* = 40	78 (70–85), *N* = 46	**<0.001**
**Group characteristics at study visit**			
Age ^(years)^	33 (30–36.75), *N* = 52	33 (29–36), *N* = 49	0.663
BMI ^(kg/m^2^)^	29.14 (24.86–36.89), *N* = 51	24.98 (22.83–28.12), *N* = 47	**<0.001**
Gravida	1 (1–2.5), *N* = 49	1.5 (1–2), *N* = 42	0.733
Para	1 (1–2), *N* = 49	1 (1–2), *N* = 42	0.961
BP systolic ^(mmHg)^	131 (126–145), *N* = 51	114 (109–124), *N* = 49	**<0.001**
BP diastolic ^(mmHg)^	91 (84–97), *N* = 51	74 (68–82.5), *N* = 49	**<0.001**
Antihypertensive Medication ^at study visit^	37 (78.7), *N* = 47	0 (0), *N* = 49	**<0.001**
IPAQ ^4^			
Low physical activity	35 (67.3), *N* = 52	32 (65.3), *N* = 49	0.946
Moderate physical activity	12 (23.1), *N* = 52	10 (20.4), *N* = 49	0.788
High physical activity	5 (9.6), *N* = 52	6 (12.2), *N* = 49	0.647
Consumption behavior			
Smoking	2 (5), *N* = 40	4 (10.8), *N* = 37	0.345
Caffeine ^(within the last 4 h)^	8 (30.8), *N* = 26	4 (18.2), *N* = 22	0.321
Nutrition			
Fruit and vegetables ^(in portions)^	3 (2–4), *N* = 23	3 (2–4), *N* = 20	0.910
Salt intake ^(in teaspoons)^	3 (2–3), *N* = 23	3 (2–3), *N* = 19	0.827
Sugary drinks ^(in glasses)^	1 (1–3), *N* = 23	1 (1–1), *N* = 20	**0.038**
Wholemeal products ^(in portions)^	2 (1–3), *N* = 23	2 (1–2.75), *N* = 20	0.897
Socio-economic status			
Highest school qualification			0.284
No school-leaving qualification	0 (0), *N* = 23	0 (0), *N* = 20	
A-levels	14 (60.9), *N* = 23	16 (80), *N* = 20	
secondary school	6 (26.1), *N* = 23	4 (20), *N* = 20	
lower secondary school	2 (8.7), *N* = 23	0 (0), *N* = 20	
Others	1 (4.3), *N* = 23	0 (0), *N* = 20	

Data are *N*/*n* (%) or median (25th–75th percentile). Significant results by Mann–Whitney U test (*p*  <  0.05) are highlighted in bold; ^1^ current intake of antihypertensive medication and/or blood pressure levels ≥ 140/90 mmHg at study visit; ^2^ high blood pressure before pregnancy or in the first trimester of pregnancy; ^3^ cardiovascular diseases (heart valve regurgitation, dilated cardiomyopathy), vascular diseases (medical history of deep vein thrombosis), diabetes (type 1 diabetes mellitus, type 2 diabetes mellitus), psychiatric disorders (anxiety disorder, depression), thyroid diseases (Hashimoto, hypothyroidism), neurologic disorders (history of stroke, epilepsy), others (bronchial asthma, chronic hepatitis B infection, Crohn’s disease, factor V mutation, alopecia, allergies, polycystic ovaries, gastro-esophageal reflux, obesity, dwarfism, hip dysplasia, endometriosis, pancreatitis, history of cancer, nephrotic syndrome, prothrombin mutation, ulcerative colitis); PE, preeclampsia; FGR, fetal growth restriction; BP, blood pressure; BMI, body mass index; ^4^ IPAQ high physical activity, vigorous intensity activity on at least 3 days achieving a minimum total physical activity of at least 1500 MET minutes a week or 7 or more days of any combination of walking, moderate intensity or vigorous intensity activities achieving a minimum total physical activity of at least 3000 MET minutes a week; IPAQ moderate physical activity, 3 or more days of vigorous intensity activity and/or walking of at least 30 min per day or 5 or more days of moderate intensity activity and/or walking of at least 30 min per day or 5 or more days of any combination of walking, moderate intensity or vigorous intensity activities achieving a minimum total physical activity of at least 600 MET minutes a week; IPAQ low physical activity, if not meeting any of the criteria for either moderate or high levels of physical activity; IPAQ, International Physical Activity Questionnaire.

**Table 2:** **Group characteristics 6 months postpartum**.

	Women with Hypertension ^1^ (*N* = 27)	Women Without Hypertension ^1^ (*N* = 36)	*p*
**Group Characteristics Prior to Pregnancy**			
Chronic diseases prior to pregnancy			
Arterial hypertension ^2^	14 (51.9), *N* = 27	3 (9.1), *N* = 33	**<0.001**
Other Diseases ^3^	12 (46.2), *N* = 26	11 (31.4) *N* = 35	0.244
**Group characteristics during pregnancy**			
Maternal age at delivery ^(years)^	33.0 (30.5–36), *N* = 26	32.0 (29.25–34.75), *N* = 36	0.465
Maternal BMI prior to pregnancy ^(kg/m^2^)^	27.58 (23.66–36.36), *N* = 26	22.27 (21.05–25.51), *N* = 35	**<0.001**
Weeks of gestation ^(weeks)^	35.5 (33–37), *N* = 26	37.0 (35.0–38.0), *N* = 36	**0.020**
Child birth weight ^(g)^	2252.50 (1706.25–2691.25), *N* = 26	2555.0 (2040.0–3012.50), *N* = 30	0.094
Twin Pregnancies	4 (14.8), *N* = 27	0 (0), *N* = 36	
Presentation of pregnancy complication PE with FGR	9 (33.3), *N* = 27	10 (27.8), *N* = 36	0.637
Presentation of pregnancy complication PE without FGR	16 (59.3), *N* = 27	13 (36.1), *N* = 36	0.070
Presentation of pregnancy complication FGR without PE	2 (7.4), *N* = 27	13 (36.1), *N* = 36	**0.009**
Medication intake during pregnancy			
Antihypertensive medication	25 (92.6), *N* = 27	17 (47.2), *N* = 36	**<0.001**
BP during pregnancy			
BP systolic 1st trimester ^(mmHg)^	134 (130–140.75), *N* = 21	116.25 (110–126.5) *N* = 28	**<0.001**
BP diastolic 1st trimester ^(mmHg)^	90 (78.75–95), *N* = 21	74.5 (69–80), *N* = 28	**<0.001**
BP systolic 2nd trimester ^(mmHg)^	136 (125–140), *N* = 23	110 (107.13–131.13), *N* = 32	**<0.001**
BP diastolic 2nd trimester ^(mmHg)^	87.50 (80–92), *N* = 23	72.25 (65–82.25), *N* = 32	**<0.001**
BP systolic 3rd trimester ^(mmHg)^	144.25 (137.63–148.88), *N* = 24	131.75 (119–139.63) *N* = 34	**<0.001**
BP diastolic 3rd trimester ^(mmHg)^	92.50 (84.63–99.25), *N* = 24	81.75 (74.38–87.13), *N* = 34	**<0.001**
**Group characteristics at study visit**			
Age ^(years)^	34 (32–36), *N* = 27	32.50 (30.0–35.75), *N* = 36	0.283
BMI ^(kg/m^2^)^	31.52 (26.22–37.65), *N* = 27	23.73 (21.01–27.29), *N* = 36	**<0.001**
Gravida	1 (1–2.25), *N* = 26	2 (1–2), *N* = 31	0.820
Para	1 (1–2.25), *N* = 26	1 (1–2), *N* = 31	0.895
BP systolic ^(mmHg)^	132 (129–141), *N* = 27	119.0 (112.0–126.75), *N* = 36	**<0.001**
BP diastolic ^(mmHg)^	93 (86–95), *N* = 27	78.0 (72.0–82.50), *N* = 36	**<0.001**
Antihypertensive Medication ^at study visit^	19 (73.1), *N* = 26	0 (0), *N* = 36	**<0.001**
IPAQ ^4^			
Low physical activity	19 (73.1), *N* = 26	27 (75), *N* = 36	0.865
Moderate physical activity	6 (23.1), *N* = 26	6 (16.7), *N* = 36	0.532
High physical activity	1 (3.8), *N* = 26	3 (8.3), *N* = 36	0.481
Consumption behavior			
Smoking	2 (8.3), *N* = 24	2 (7.4), *N* = 27	0.903
Caffeine ^(within the last 4 h)^	5 (33.3), *N* = 15	5 (25), *N* = 20	0.595
Nutrition			
Fruit and vegetables ^(in portions)^	3 (2–3.25), *N* = 10	2.5 (1.25–4), *N* = 12	0.628
Salt intake ^(in teaspoons)^	2 (2–3), *N* = 10	3 (2–3), *N* = 12	0.628
Sugary drinks ^(in glasses)^	2 (1–2.25), *N* = 10	1 (1–1), *N* = 12	0.159
Wholemeal products ^(in portions)^	2 (2–3), *N* = 10	1.5 (1–2), *N* = 12	0.314
Socio-economic status			
Highest school qualification			0.449
No school-leaving qualification			
A-levels	6 (54.5), *N* = 11	9 (75), *N* = 12	
secondary school	4 (36.4), *N* = 11	2 (16.7), *N* = 12	
lower secondary school	0 (0), *N* = 11	1 (8.3), *N* = 12	
Others	1 (9.1), *N* = 11	0 (0), *N* = 12	

Data are N/n (%) or median (25th–75th percentile). Significant results by Mann–Whitney U test (*p*  <  0.05) are highlighted in bold; ^1^ current intake of antihypertensive medication and/or blood pressure levels ≥ 140/90 mmHg at study visit; ^2^ high blood pressure before pregnancy or in the first trimester of pregnancy; ^3^ cardiovascular diseases (heart valve regurgitation, dilated cardiomyopathy), vascular diseases (medical history of deep vein thrombosis), diabetes (type 1 diabetes mellitus, type 2 diabetes mellitus), psychiatric disorders (anxiety disorder, depression), thyroid diseases (Hashimoto, hypothyroidism), neurologic disorders (history of stroke, epilepsy), others (bronchial asthma, chronic hepatitis B infection, Crohn’s disease, factor V mutation, alopecia, allergies, polycystic ovaries, gastro-esophageal reflux, obesity, dwarfism, hip dysplasia, endometriosis, pancreatitis, history of cancer, nephrotic syndrome, prothrombin mutation, ulcerative colitis); PE, preeclampsia; FGR, fetal growth restriction; BP, blood pressure; BMI, body mass index; ^4^ IPAQ high physical activity, vigorous intensity activity on at least 3 days achieving a minimum total physical activity of at least 1500 MET minutes a week or 7 or more days of any combination of walking, moderate intensity or vigorous intensity activities achieving a minimum total physical activity of at least 3000 MET minutes a week; IPAQ moderate physical activity, 3 or more days of vigorous intensity activity and/or walking of at least 30 min per day or 5 or more days of moderate intensity activity and/or walking of at least 30 min per day or 5 or more days of any combination of walking, moderate intensity or vigorous intensity activities achieving a minimum total physical activity of at least 600 MET minutes a week; IPAQ low physical activity, if not meeting any of the criteria for either moderate or high levels of physical activity; IPAQ, International Physical Activity Questionnaire.

**Table 3:** **Results of cardiovascular function analysis via VICORDER**.

6 Weeks Postpartum			
	Women with Hypertension ^1^ (*N* = 52)	Women Without Hypertension ^1^ (*N* = 49)	*p*
PP ^(mmHg)^	42.5 (38–49.25), *N* = 42	38 (34–42.5), *N* = 40	**0.010**
PWV ^(m/s)^	7.4 (7–8.1), *N* = 45	6.7 (6.25- 7.25), *N* = 39	**<0.001**
Aix ^(%)^	22.5 (18.5–25), *N* = 43	19.5 (16.63–23.25), *N* = 40	0.180
AoPP ^(mmHg)^	40.5 (36–47.38), *N* = 42	36.25 (32.63–40.25), *N* = 40	**0.007**
AoBP sys ^(mmHg)^	129.75 (122.25–138.38), *N* = 42	111.75 (106.75–120.88), *N* = 40	**<0.001**
MAP ^(mmHg)^	109.25 (103.38–116.63), *N* = 42	93 (89.1–101.38), *N* = 40	**<0.001**
SV ^(mL)^	72.25 (64.13–88.13), *N* = 42	69 (62.38–72), *N* = 40	0.054
CO ^(L/min)^	4.89 (4.3–5.82), *N* = 42	4.28 (3.74–4.92), *N* = 40	**0.010**
CI ^(L/min/m^2^)^	2.52 (2.18–3.47), *N* = 42	2.4(1.98–2,75), *N* = 40	0.159
SEVR ^(%)^	166.25 (141.38–189.88), *N* = 42	165 (146.25–199.25), *N* = 40	0.591
TPR ^(PRU)^	1.32 (1.16–1.56), *N* = 43	1.28 (1.09–1.57), *N* = 40	0.909
FMS ^(%)^	18 (9.75–24), *N* = 46	17 (14–23), *N* = 51	0.597
**6 months postpartum**			
	**Women with hypertension** ^1^ **(*N* = 27)**	**Women without hypertension** ^1^ **(*N* = 36)**	***p***
PP ^(mmHg)^	43 (37–47), *N* = 25	40.5 (36.875–48.25), *N* = 34	0.830
PWV ^(m/s)^	7.2 (6.66–7.59), *N* = 24	6.73 (6.34- 7.46), *N* = 34	0.053
Aix ^(%)^	20.5 (16.75–26), *N* = 25	19 (15.5–23.25), *N* = 34	0.149
AoPP ^(mmHg)^	40.5 (30–45), *N* = 25	38.25 (35–44.38), *N* = 34	0.741
AoBP sys ^(mmHg)^	128 (124.5–134.5), *N* = 25	116.25 (110.38–124.25), *N* = 34	**<0.001**
MAP ^(mmHg)^	110 (104.75–114), *N* = 25	98.5 (92–101.625), *N* = 34	**<0.001**
SV ^(mL)^	70 (58.5–76.75), *N* = 25	69.75 (65.88–87.63), *N* = 34	0.490
CO ^(L/min)^	4.74 (4.02–5.12), *N* = 25	4.97 (4.07–5.88), *N* = 34	0.145
CI ^(L/min/m^2^)^	2.48 (2.00–2.73), *N* = 25	2.90(2.26–3.21), *N* = 34	**0.013**
SEVR ^(%)^	185 (155–201), *N* = 25	163 (145.25–179.13), *N* = 34	0.071
TPR ^(PRU)^	1.39 (1.27–1.71), *N* = 25	1.14 (1.01–1.41), *N* = 34	**<0.001**
FMS ^(%)^	14 (10.5–20), *N* = 25	19.5 (12.75–25.25), *N* = 34	0.094

Data are N/n (%) or median (25th-75th percentile). Number of subjects (N) is given if deviating from indicated group size. Significant results by Mann–Whitney U test (*p*  <  0.05) are highlighted in bold. ^1^ Current intake of antihypertensive medication and/or blood pressure values ≥ 140/90 mmHg at study visit; PP, pulse pressure; PWV, pulse wave velocity; AoPP, aortic pulse pressure; AoBP sys, aortic blood pressure systolic; MAP, mean arterial pressure; SV, stroke volume; CO, cardiac output; CI, cardiac index; Aix, augmentation index; SEVR, subendocardial viability ratio; TPR, total peripheral resistance; FMS, flow-mediated slowing.

**Table 4:** **Adjusted odds ratios (aORs) for arterial hypertension 6 weeks and 6 months after pregnancy**.

	Arterial Hypertension 6 Weeks Postpartum			Arterial Hypertension 6 Months Postpartum		
	aOR ^2^ (CI ^3^)	*p*	Nagelkerke’s R-Squared	aOR (CI)	*p*	Nagelkerke’s R-Squared
BP ^1^ systolic 1st ^trimester (mmHg)^	1.82 (1.12–2.97)	**0.016**	0.456	2.57 (1.32–5.02)	**0.005**	0.566
BP diastolic 1st ^trimester (mmHg)^	2.50 (1.33–4.68)	**0.004**	0.503	3.39 (1.42–8.12)	**0.006**	0.566
BP systolic 2nd ^trimester (mmHg)^	2.76 (1.69–4.56)	**<0.001**	0.486	2.46 (1.33–4.57)	**0.004**	0.519
BP diastolic 2nd ^trimester (mmHg)^	3.42 (1.84–6.35)	**<0.001**	0.451	5.51 (1.86–16.43)	**0.002**	0.590
BP systolic 3rd ^trimester (mmHg)^	1.33 (0.86–2.08)	0.198	0.432	2.18 (1.01–4.68)	**0.047**	0.575
BP diastolic 3rd ^trimester (mmHg)^	1.84 (1.01–3.36)	**0.048**	0.429	4.68 (1.71–12.96)	**0.003**	0.609

Adjustments were made for maternal BMI prior to pregnancy, antihypertensive medication intake during pregnancy, chronic hypertension prior to pregnancy, presentation of pregnancy complications preeclampsia and/or fetal growth restriction, blood pressure values for every trimester of pregnancy; significant results (*p*  <  0.05) are highlighted in bold; odds ratios and confidence intervals are given for each 10 mmHg increase in blood pressure, ^1^ BP, blood pressure; ^2^ aOR, adjusted odds ratio; ^3^ CI, confidence intervals.

## Data Availability

The original contributions presented in this study are included in the article. Further inquiries can be directed to the corresponding author.

## Supplementary Materials

The following supporting information can be downloaded at: https://www.mdpi.com/article/10.3390/jcm15124646/s1, Variables from the final step of the binary logistic regression (Wald backward method) for each trimester.
