# Supplementary material for: Impact of Blood Pressure Levels During Pregnancy on Postpartum Hypertensive Outcomes: Insights from a Cohort Study
Source: J Clin Med. 2026 Jun 15;15(12):4646. doi: 10.3390/jcm15124646 (PMC13302391; doi:10.3390/jcm15124646)
Supplement: Supplementary file 1 [file jcm-15-04646-s001.zip › jcm-4293434-supplementary.pdf]

Supplement

Impact of Blood Pressure Levels During Pregnancy on Postpartum Hypertensive Outcomes: Insights from a Cohort Study

Anne-Christin Loheit [1,*,†,‡], Charlotte Lößner [2,†], Yvonne Lindemann [1], Ekkehard Schleussner [1,‡] and Tanja Groten [3,‡]

1 Department of Obstetrics, University Hospital Jena, Friedrich-Schiller-University Jena, Am Klinikum 1, 07747 Jena, Germany
2 Department of Gynecology, University Hospital Jena, Friedrich-Schiller-University Jena, Am Klinikum 1, 07747 Jena, Germany
3 Department of Obstetrics, University of Cologne, Faculty of Medicine and University Hospital Cologne, 50931 Cologne, Germany
* Correspondence: anne-christin.loheit@med.uni-jena.de
† These authors contributed equally to this work and share first authorship.
‡ Member of the Center for Early Pregnancy and Reproductive Health (CEPRE).

Variables from the final step of the binary logistic regression (Wald backward method) for each trimester

	**Arterial hypertension 6 weeks postpartum visit**			**Arterial hypertension 6 months postpartum visit**		
	Variable	OR[2] (CI)	p	Variable	OR (CI)	p
BP[1] systolic 1st trimester (mmHg)	maternal BMI prior to pregnancy	1.11 (0.99-1.23)	0.070	maternal BMI prior to pregnancy	1.19 (1.02-1.38)	0.025
	antihypertensive medication intake during pregnancy	3.41 (0.91-12.80)	0.069			
BP diastolic 1st trimester (mmHg)	maternal BMI prior to pregnancy	1.11 (0.99-1.24)	0.076	maternal BMI prior to pregnancy	1.24 (1.05-1.46)	0.011
	antihypertensive medication intake during pregnancy	4.01 (1.07-15.11)	0.040			
BP systolic 2nd trimester (mmHg)	fetal growth restriction	0.22 (0.04-1.22)	0.083	maternal BMI prior to pregnancy	1.14 (0.99-1.30)	0.075
	preeclampsia and fetal growth restriction	0.26 (0.70-0.99)	0.048			
BP diastolic 2nd trimester (mmHg)	fetal growth restriction	0.25 (0.06-1.10)	0.067	maternal BMI prior to pregnancy	1.15 (0.99-1.33)	0.065
BP systolic 3rd trimester (mmHg)	maternal BMI prior to pregnancy	1.12 (1.01-1.25)	0.026	maternal BMI prior to pregnancy	1.23 (1.06-1.42)	0.005
	chronic hypertension prior to pregnancy	2.92 (0.83-10.35)	0.096	antihypertensive medication intake during pregnancy	5.28 (0.77-36.18)	0.090
	antihypertensive medication intake during pregnancy	5.55 (1.73-17.88)	0.004			

BP diastolic 3rd trimester (mmHg)	maternal BMI prior to pregnancy<br><br>antihypertensive medication intake during pregnancy	1.12 (1.02-1.24)<br><br>3.85 (1.10-13.47)	0.020<br><br>0.035	maternal BMI prior to pregnancy	1.27 (1.10-1.47)	0.001
Significant results $p < 0.05$; [1]BP, blood pressure; [2]OR, Odds Ratio						
